# Cannabidiol (CBD) for Treatment of Neurofibromatosis-related Pain and Concomitant Mood Disorder: A Case Report

**DOI:** 10.7759/cureus.6312

**Published:** 2019-12-06

**Authors:** Omar Hegazy, Howard Platnick

**Affiliations:** 1 Family Medicine, Family Practice, Toronto, CAN; 2 Family Medicine/Chronic Pain and Medical Cannabis, Family Practice, Toronto, CAN

**Keywords:** depression, neurofibromatosis type 1 (nf1), cannabidiol, pain, anxiety

## Abstract

Neurofibromatosis type 1 (NF1) is a common genetic disorder. Pain is a major symptom of this disease which can be secondary to the development of plexiform and subcutaneous neurofibromas, musculoskeletal symptoms (such as scoliosis and pseudoarthrosis), and headaches. Visible neurofibromas add significant psychosocial distress for NF1 patients. Along with the chronic pain, psychosocial distress contributes to associated mood disorders, such as depression and anxiety. Cannabis has been the focus of many studies for treating multiple conditions, including epilepsy, multiple sclerosis, Parkinsonism disease, and many chronic pain conditions. Cannabidiol (CBD) is the major non-psychotropic component of cannabis. CBD has shown anti-inflammatory and analgesic properties, as well as having mood stabilizer and anxiolytic effects. In this report, we present the use of cannabidiol (CBD) for the management of chronic pain and concomitant mood disorder in an NF1 patient.

## Introduction

Neurofibromatosis type 1 (NF1) is one of the most common autosomal dominant genetic disorders, affecting approximately 1 in 2,600 to 3,000 people [1]. A mutation in the NF1 gene on chromosome 17 causes a loss of function or production of the neurofibromin protein, encoded by the gene. Clinically, NF1 is characterized by the presence of multiple café-au-lait spots, Lisch nodules of the iris, and neurofibromas. However, the widespread expression of the neurofibromin protein throughout the body and the variability in mutation expression involves multiple body systems that can vary greatly from one patient to another, between families, and even within a given family carrying the same mutation [2]. These patients have a high predisposition for developing benign and malignant tumors due to the loss of the tumor suppression property of the NF1 gene. More commonly, they can develop cutaneous and subcutaneous neurofibromas, gliomas of the optic pathway, malignant peripheral nerve sheath tumors, and gastrointestinal stromal tumors [3]. Chronic pain is commonly associated with this condition, mainly due to the development of these tumors, notably plexiform and subcutaneous neurofibromas, musculoskeletal symptoms (such as scoliosis and pseudoarthrosis), and headaches. These patients do not only suffer from physical distress but the accompanying body dysmorphia also affects their psychological well-being and overall quality of life [4]. In prior studies, the prevalence of pain among patients with NF1 ranged from 55% to 70%, with 35% of patients complaining of chronic pain [4-5]. More importantly, pain chronicity is related to mental health problems, including depression and anxiety [4]. These symptoms are worse when the disease presents on the face or other visible regions of the body. This leads to patients reporting increased psychological burdens related to issues of self-perception, drastically reducing their overall quality of life [5-6]. Although pain is clearly a frequent problem in NF1, with 41% of the patient’s reporting use of over-the-counter (OTC) or prescribed pain medications, it is still understudied and underreported in the medical literature [4, 7]

The goal of this case report is to demonstrate and present the use of cannabidiol (CBD), one of the major components of phytocannabinoids (plant cannabinoid), as a treatment option for managing NF1-related pain, anxiety, and depression, and increasing the overall quality of life in these patients.

## Case presentation

A 25-year-old woman of African descent with NF1 presented to our clinic complaining of chronic pain, depression, and anxiety. She was diagnosed with NF1 at puberty but had reported symptoms since childhood. She was unaware of a family history of similar conditions. She has Lisch nodules on her iris bilaterally and 10 café-au-lait patches across her body. The patient also has numerous subcutaneous neurofibromas on her face (plainly visible and causing some facial disfigurement) and in several regions of her body. These caused her chronic, intermittent, sharp pain of varying intensity but averaging 6/10 on the 10-point pain scale. She has tried many OTC pain medications with limited benefits. She has had over 20 surgical resections and multiple laser treatments for the neurofibromas with limited success as they often recurred after treatment. She reports pain from both the treatment and the tumors themselves. The neurofibroma tumors have also caused her cosmetic issues. She reports being withdrawn, stressed, and anxious, especially in social situations when she feels that people are staring at her. She reports low energy and had lost interest in most activities. Furthermore, she reports that her condition has interfered with finding employment and leading a normal life. She states that her mood is low, and she has frequent crying episodes. Her general practitioner (GP) has prescribed multiple antidepressants that were not tolerated due to the side effects. Consistent with NF1 common symptoms, she reports having migraine headaches. She averages around 15 episodes per month varying in intensity between 5/10 and 10/10. She has reportedly used OTC pain medications for the migraines unsuccessfully.

CBD oil has no established dosing guidelines or maximum doses, except in psychosis (800 mg) and seizure disorders (2,500 mg or 25 - 50 mg/kg) [8]. Dosing is highly individualized and relies to a great extent on titration. The general approach to initiation is to "start low and go slow." Many patients report benefits at low doses, starting with as little as 5 - 20 mg (0.25 ml - 1 ml) per day of oral oil preparations [8].

The risks and benefits of using cannabis for her condition were discussed in detail. She was advised to start a three month trial of sublingual CBD oil at 4 mg (0.2 ml) twice per day (BID) and titrate upwards to 10 mg (0.5 ml) BID with a maximum increase up to 20 mg (1 ml) BID if no response. She was advised to try and hold it in contact with the buccal membrane for at least 90 seconds [9]. The recommended formulation had low amounts of tetrahydrocannabinol (THC) at less than 1 mg per 1 ml of oil. The low levels of THC reportedly assist CBD in reaching the receptors, having a synergistic benefit [10].

At the three-month follow-up visit, the patient reported starting with 4 mg (0.2 ml) BID of cannabis oil with CBD to THC ratio concentrations of 20 mg/ml to 1 mg/ml (CBD: THC 20:1). This was gradually increased by the patient (self-titration) to 8 mg (0.4 ml) BID. During this period, the patient reported that her pain was significantly reduced (from an average of 6/10 down to 1/10). She reported being less emotional and feeling calmer. She said that her anxiety dropped from 9/10 down to 3 to 4/10 and that her mood had stabilized. Also, as an added benefit, she reported that her migraines had improved. She reported fewer episodes per month (five per month down from 15 per month before starting the CBD oil) and that she was able to decrease her dependence on OTC anti-inflammatory pills for pain relief. Overall, a significant improvement in her quality of life was evident by the change in her demeanor and a novel enthusiasm for seeking employment.

CBD is generally better tolerated by patients when compared to THC. Some patients may experience side effects, usually mild and infrequent, including fatigue, diarrhea, increased/decreased appetite, and somnolence [11]. These may be due to the low levels of THC in the formulation [12]. Our patient did not report any side effects during the treatment period.

## Discussion

Cannabidiol (CBD) is the major non-psychotropic component of phytocannabinoids extracted from the Cannabis sativa plant. CBD interacts with the human endocannabinoid system mainly through two G-protein-coupled membrane receptors - cannabinoid receptor 1 (CB1) and cannabinoid receptor 2 (CB2). Predominantly, CB1 receptors are located in the central and peripheral nervous system, while CB2 receptors are found in immune cells. THC acts as an inverse agonist on these receptors, while CBD exhibits antagonist properties on them. Specifically, this antagonism on CB2 receptors inhibits immune cell migration and might explain the anti-inflammatory effects of CBD, as well as the attenuation of THC psychoactive side-effects [13]. Recent studies have also shown that CBD can interact with a wide range of other targets, such as sodium, potassium, and calcium channels, and serotonin and glycine receptors, independently from the CB1 and CB2 receptors [14].

CBD has been showing positive results in alleviating chronic pain. A large meta-analysis by Whiting et al. and other studies showed that cannabinoids are effective with neuropathic pain, fibromyalgia, cancer, and diabetic neuropathy, refractory pain due to multiple sclerosis and other neurological conditions, including rheumatoid arthritis, noncancer pain, central pain, musculoskeletal problems, and chemotherapy-induced pain [15-16]. Its efficacy with migraines and mood disorders has been consistently noted in the literature as well [17-19]. A recent preclinical study demonstrated effective neuropathic pain and comorbid anxiety and depression reduction through its interaction with the serotonin 5-HT_1A _receptor [20]. It is speculated that this might be the physiological model responsible for the noted improvement in the case study. Similarly, a study on the mechanism of pain in NF1 patients proposes that modulation of ion channels, specifically sodium channels, might be one of the pain pathways in these patients [7]. Giving the effect of CBD on these channels, it is postulated that the CBD might be modulating pain via this mechanism, as described above. 

Current treatment options for pain in NF1 include OTC medications (such as ibuprofen and acetaminophen), prescribed pain medications (such as opioids, anticonvulsants, and anti-depressants), and surgical removal of neurofibromas. Other than emerging potential targeted therapies, specific treatments for NF1 are rather deficient due to the complex nature of the NF1 pathophysiology and the minimal understanding of its pain etiology [7].

## Conclusions

Cannabidiol use with our patient has shown promising results in controlling the pain and concomitant mood disorder. Given our experience and the growing body of evidence for the efficacy of CBD in treating patients with multiple chronic pain conditions, it should be considered as a treatment option in the management of this patient group, especially when conventional pain medications have failed or were not well-tolerated. CBD also seems to have a positive effect on the psychosocial pain component of NF1. Nevertheless, more research targeted at understanding the etiology of the pain in NF1 and also more clinical trials using CBD for NF1 are still needed.
